# A Paleolithic diet lowers resistant starch intake but does not affect serum trimethylamine-*N*-oxide concentrations in healthy women

**DOI:** 10.1017/S000711451800329X

**Published:** 2018-12-17

**Authors:** Angela Genoni, Johnny Lo, Philippa Lyons-Wall, Mary C. Boyce, Claus T. Christophersen, Anthony Bird, Amanda Devine

**Affiliations:** 1 School of Medical and Health Sciences, Edith Cowan University, Joondalup, WA 6027, Australia; 2 School of Science, Edith Cowan University, Joondalup, WA 6027, Australia; 3 School of Molecular and Life Sciences, Curtin University, Bentley, WA 6102, Australia; 4 CSIRO Health & Biosecurity, Gate 13, Kintore Avenue, Adelaide, SA 5000, Australia

**Keywords:** Paleolithic diet, Resistant starch, Dietary fibre, Food groups

## Abstract

The Paleolithic diet excludes two major sources of fibre, grains and legumes. However, it is not known whether this results in changes to resistant starch (RS) consumption. Serum trimethylamine-*N*-oxide (TMAO) is produced mainly from colonic fermentation and hepatic conversion of animal protein and is implicated in CVD, but changes in RS intake may alter concentrations. We aimed to determine whether intake of RS and serum concentrations of TMAO varied in response to either the Paleolithic or the Australian Guide to Healthy Eating (AGHE) diets and whether this was related to changes in food group consumption. A total of thirty-nine women (mean age 47 (sd 13) years, BMI 27 (sd 4) kg/m^2^) were randomised to AGHE (*n* 17) or Paleolithic diets (*n* 22) for 4 weeks. Serum TMAO concentrations were measured using liquid chromatography–MS; food groups, fibre and RS intake were estimated from weighed food records. The change in TMAO concentrations between groups (Paleolithic 3·39 μm *v*. AGHE 1·19 μm, *P* = 0·654) did not reach significance despite greater red meat and egg consumption in the Paleolithic group (0·65 serves/d; 95 % CI 0·2, 1·1; *P* <0·01, and 0·22 serves/d; 95 % CI 0·1, 0·4, *P* <0·05, respectively). RS intake was significantly lower on the Paleolithic diet (*P* <0·01) and was not associated with TMAO concentrations. However, the limited data for RS and the small sample size may have influenced these findings. While there were no significant changes in TMAO concentrations, increased meat consumption and reduced RS intake warrant further research to examine the markers of gastrointestinal health of Paleolithic diet followers and to update Australian food databases to include additional fibre components.

Previous research on the Paleolithic diet has examined its impact on cardiovascular, anthropometric and metabolic outcomes^(^ ^1^ ^–^ ^9^ ^)^. The elimination of cereals, legumes and dairy products from the Paleolithic diet means food patterns are likely to change significantly, yet there is little research on the changes in intake from the core food groups^(^ ^4^ ^,^ ^5^ ^,^ ^10^ ^)^. Although dietary fibre intake on a Paleolithic diet is often not significantly different, when compared with a control or standard diet^(^ ^3^ ^–^ ^8^ ^,^ ^10^ ^)^, it has yet to be established whether the dietary fibre profile is altered. Grains and pulses are important sources of a particular type of dietary fibre, resistant starch (RS). RS offers a range of health benefits mediated through the products of its fermentation by colonic bacteria^(^ ^11^ ^)^.

Determination of RS content is problematic because levels differ depending on food preparation and cooking methods, storage conditions, level of ripeness and measurement technique^(^ ^12^ ^)^. Current estimates of RS in an Australian diet are 3·4–9·4 g/d^(^ ^13^ ^)^, but it has been suggested that increasing intake to approximately 20 g/d may be required to obtain health benefits from the prebiotic capacity^(^ ^14^ ^)^. Therefore, RS intake on a Paleolithic diet requires quantification to understand potential implications for the long-term gut and overall health outcomes.

Trimethylamine (TMA) is produced *in vivo* by the microbiota of the bowel, from consumption of choline, phosphatidylcholine and l-carnitine^(^ ^15^ ^,^ ^16^ ^)^. Choline and phosphatidylcholine are found predominately in milk, liver, meat, eggs and peanuts^(^ ^17^ ^)^, and l-carnitine are predominately found in red meat^(^ ^15^ ^)^. TMA is converted in the liver to trimethylamine-*N*-oxide (TMAO)^(^ ^18^ ^)^. Wang *et al.* ^(^ ^16^ ^)^ showed that TMAO was a dose-dependent predictor of CVD risk in a cohort of 1876 subjects and positively correlated with atherosclerotic plaque size (Pearson correlation *R* 0·42, *P* <0·001) in a rodent study. An evaluation of the prognostic value of TMAO concentrations showed that patients with heart failure had significantly higher plasma TMAO concentrations, associated with an increase in mortality (3·4 times) risk^(^ ^19^ ^)^. It is still not understood whether TMAO is a causative or correlative factor in atherosclerotic plaque development, and more research is required to understand the link with CVD. TMAO is filtered and excreted by kidneys into the urine and elevations in serum TMAO may merely be a marker of reduced kidney function, often co-existing with coronary atherosclerosis^(^ ^20^ ^)^. Similarly the increased risk of colorectal cancer associated with plasma TMAO in women^(^ ^21^ ^)^ may merely be a demonstrating that TMAO serves as a marker for red and processed meat consumption, which are already established risk factors for this disease^(^ ^22^ ^)^. Egg and red meat consumption elevate plasma TMAO concentrations in the 24 h following consumption^(^ ^20^ ^,^ ^23^ ^)^. For seafood, which is a source of pre-formed TMAO, the postprandial rise in circulating levels can be detected within 15 min of consumption^(^ ^24^ ^)^, indicating that TMAO is rapidly absorbed from the upper gut, without entering the colon^(^ ^24^ ^)^. Individual variation in gut microbiota composition may also influence the production of TMAO in response to dietary sources of choline and l-carnitine^(^ ^24^ ^)^. A recent paper reported that diets high in RS (19–27 g/d), but low in carbohydrate (CHO), were also associated with increased plasma TMAO concentrations^(^ ^25^ ^)^. This suggests that colonic CHO fermentation may play a significant role in mediating microbial populations, which produce TMA. CHO intake are restricted on the Paleolithic diet, with cereal fibres excluded; therefore, it is important to understand how this dietary pattern might influence circulating TMAO concentrations. We aimed to determine whether fasting TMAO concentrations varied in healthy subjects randomised to either the Paleolithic or the Australian Guide to Healthy Eating (AGHE) diet, for a 4-week intervention and the relationship with dietary RS, total fibre and food group intake.

## Methods

The current data were part of a larger study described elsewhere^(^ ^3^ ^)^. In brief, a 4-week dietary intervention was conducted in thirty-nine healthy females (mean age 47 (sd 13) years, BMI 27 (sd 4) kg/m^2^) using *ad libitum* Paleolithic (*n* 22) and AGHE diets (*n* 17), with 3-d weighed food records collected pre- and post-intervention^(^ ^23^ ^)^. A priori sample size calculations were based on an expected 15 % reduction in the primary outcome variable, total cholesterol, with 5 % *α*-error and 80 % power between groups. Participants were locally recruited from areas surrounding the university campus. The study was approved by the Edith Cowan Human Research Ethics Committee (project 13402) and registered on the Australian and New Zealand Register of Clinical Trials (ACTRN12615000246583).

### Food group intake

To assess food group intake, the pre- and post-intervention 3-d weighed food records collected previously^(^ ^23^ ^)^ were re-analysed using Australian software, FoodWorks Professional, version 8.0^(^ ^26^ ^)^, with food group analysis enabled to provide intake of the major food groups: grains, fruits, vegetables, protein foods and dairy foods and intake of fats, oils and added sugars. There were 1211 individual food items utilised for the 78, 3-d food records analysed. A local copy of the database was made, and within FoodWorks, each food item was updated to the equivalent food item in the AUSFoods 2015^(^ ^26^ ^)^ and AUSNUT 2013^(^ ^27^ ^)^ databases. To assess fibre fraction intake, an additional local copy of the database was made, with the NZFoodFiles 2014 data set enabled^(^ ^28^ ^)^. Each food item was updated with soluble and insoluble fibre content. Where data were missing from FoodFiles 2014^(^ ^29^ ^)^, or there was no appropriate match with the consumed food, soluble and insoluble fibre data were sourced from published literature^(^ ^30^ ^–^ ^32^ ^)^. Minimum and maximum estimations were utilised for estimation of RS content based on data published by Roberts *et al.* ^(^ ^13^ ^)^. All food items in the database were updated with an RS value. Similar foods were grouped, and RS content was assumed to be consistent across the category. For example, estimations of RS content are available for wholemeal, white, rye and flatbreads^(^ ^13^ ^)^. Therefore, breads were grouped into one of these categories, and RS content was assumed to be consistent across the category. Foods for which no data were available were assigned a value of 0. Fibre and RS content of mixed dishes and recipes were determined based on fibre/RS proportions of individual ingredients within the recipe.

### Trimethylamine-*N*-oxide analysis

TMAO analysis was conducted on fasting serum samples stored at –20°C and collected as part of the study as described previously^(^ ^3^ ^)^. Serum was thawed, and 150 μl was transferred to a 2-ml Eppendorf tube. Liquid chromatography (LC)–MS grade acetonitrile (450 μl), containing 0·1 μg/ml of deuterated TMAO (TMAO-d^9^) as the internal standard, was added to the serum. The tube was vortexed for 30 s and centrifuged at 4000 rpm and 4°C for 10 min. An aliquot (100 μl) of the supernatant was transferred to a 2-ml HPLC vial, diluted with 75:25 acetonitrile containing 0.1 % formic acid: 100 mm ammonium formate (400 μl) and mixed thoroughly. Calibration standards in the range of 0·05–5 μg/ml TMAO were prepared in 0·1 % formic acid and check standards (5, 0·25 and 0·05 μg/ml TMAO) were prepared in synthetic serum and then processed using the same procedure as for the samples. The calibration standards, check standards and samples were analysed by LC–MS/MS using a Thermo Scientific Ultimate 3000 Liquid Chromatograph and coupled to a Thermo Scientific Quantiva triple quadrupole mass spectrometer. The LC separation was achieved using an Intrada amino acid column (150 mm × 3·0 μm inner diameter) packed with 3 μm particles. Aliquots (2 μl) were injected into the column and separated using a mobile phase of acetonitrile with 0·1 % formic acid (A) and 100 mm ammonium formate (B). Initial mobile phase conditions were 75 % A and 25 % B held for 3 min, then B was increased to 100 % over 4 min and maintained for 3 min, and B was reduced to 25 % over 0·1 min and maintained for 2 min. The flow rate was 0·6 ml/min, and the column was maintained at 35°C. Detection was performed in positive mode (3500 V), and the analytes were ionised by electrospray and monitored in multiple reaction monitoring mode. The MS was operated under the following conditions: gases (arbitrary units) sheath 52, auxiliary 16 and sweep 2; ion transfer tube temperature was 356°C; vapouriser temperature 420°C; transitions of *m*/*z* 76·2 to *m*/*z* 48·2, 58·1 and 59·2 with collision energies (eV) of 30, 16 and 11, respectively, were used to monitor TMAO; transitions of *m*/*z* 85·2 to *m*/*z* 46·2, 66·2 and 68·2 with collision energies (eV) of 41, 21 and 11, respectively, were used to monitor TMAO-d^9^.

### Statistical analyses

Statistical analyses were conducted using SPSS for Windows, version 23^(^ ^33^ ^)^. Pre- and post-intervention data were examined for normality using the Shapiro–Wilk test. Independent *t* test and Mann–Whitney *U* test for non-normally distributed data were performed to examine the differences between the two groups in each of the dietary intake variables. Paired *t* test and Wilcoxon signed-rank test were conducted to determine the changes within groups. ANCOVA was used to examine the associations between TMAO concentrations and changes in dietary variables, with particular attention to protein and meat servings, egg, solid fat and grain servings, in addition to RS intake. Statistical significance was set at *P* <0·05.

## Results

### Food group serves

As expected on a Paleolithic diet, there were significant reductions in the consumption of total grains (–4·6 serves/d) and dairy products (–1·5 serves/d) (*P* <0·01 for both) (Table 1). Post-intervention, the Paleolithic group had a higher intake of protein foods (3·9 *v*. 2·6 serves/d), due to greater red meat consumption, and higher intake of fruits (3·1 *v*. 1·2 serves/d) and vegetables (5·8 *v*. 4·0 serves/d) (*P* <0·01 for all), compared with AGHE group. Whole grains contributed approximately half of the total grain intake (55·2 %) in the AGHE group. Consumption of oil, solid fat equivalents and the percentage of energy consumed as added sugars were not significantly different between groups.

**Table 1:** Daily consumption by food group pre- and post-intervention, showing within- and between-group changes† (Mean values and standard deviations; medians and interquartile ranges (IQR); medians and 95 % confidence intervals)

Food group (serve size)	Paleolithic (*n* 22)						AGHE (*n* 17)									
	Pre		Post		Change within group		Pre		Post		Change within group		Difference between groups (change)		Difference between groups post-intervention	
	Mean	sd	Mean	sd	Median	95 % CI	Mean	sd	Mean	sd	Median	95 % CI	Median	95 % CI	Median	95 % CI
Total grains	5·18	1·71	0·58	0·97	–4·60**	–5·5, –3·7	4·42	1·95	4·50	1·93	0·08	–1·1, 1·3	–4·68**	–6·1, –3·3	–3·92**	–5·0, –2·9
Refined grains (16 g starch equivalent)	3·31	1·55	0·37	0·65	–2·94**	–3·6, –2·3	2·54	1·61	2·42	1·53	–0·12	–1·2, 1·0	–2·82**	–4·0, –1·6	–2·06**	–2·9, –1·2
Whole grains (14 g starch equivalent)	1·86	1·12	0·21	0·45	–1·65**	–2·3, –1·1	1·87	1·34	2·07	1·36	0·20	–0·7, 1·1	–1·85**	–2·9, –0·9	–1·86**	–2·6, –1·1
Wholegrain percentage‡	37·11	35·16	0·00	27·59	–29·17**§	–46·1, –5·7	41·34	37·80	55·25	46·66	5·68§	–12·8, 24·1	–34·33*||	–60·1, –7·4	–31·27**||	–60·8, –22·2
Total fruit‡	1·52	0·62	3·06	1·85	1·36**§	0·9, 2·0	1·65	1·00	1·18	1·43	–0·28§	–0·7, 0·2	1·59**||	0·9, 2·3	1·53**||	0·6, 2·4
Citrus, melons, berries (fresh equivalent 350 kJ, dried 30 g)‡	0·16	0·41	0·22	0·71	0·06§	–0·1, 0·3	0·13	0·31	0·10	0·24	–0·02§	–0·1, 0·1	0·06||	–0·1, 0·3	0·11||	–0·02, 0·4
Other fruit (fresh equivalent 350 kJ, dried 30 g)‡	1·12	0·71	2·50	1·70	1·20**§	0·8, 1·8	1·62	0·87	1·18	1·38	–0·18§	–0·6, 0·4	1·36**||	0·7, 2·1	1·27**||	0·45, 1·87
Fruit juice (125 ml)‡	0·00	0·00	0·0	0·0	0·0§	0·0, 0·0	0·00	0·00	0·00	0·00	0·0§	0·0, 0·0	0·0||	0·0, 0·0	0·00||	0·00, 0·00
Fruit juice percentage‡	0·00	0·00	0·0	0·0	0·0§	0·0, 0·0	0·00	0·00	0·00	0·00	0·0§	0·0, 0·0	0·0||	0·0, 0·0	0·00||	0·00, 0·00
Total vegetables	3·51	1·10	5·76	2·77	2·25**	1·1, 3·5	4·45	2·05	4·00	2·06	–0·45	–1·7, 0·8	2·70**	1·0, 4·4	1·75*	0·1, 3·4
Dark green vegetables (100 kJ equivalent)	0·36	0·37	0·47	0·52	0·11	–0·1, 0·4	0·24	0·24	0·17	0·19	–0·07	–0·2, 0·1	0·18	–0·1, 0·5	0·29*	0·0, 0·5
Red and orange vegetables (150 kJ)‡	0·89	0·35	3·04	2·44	2·01**§	1·2, 3·1	0·83	0·87	1·33	1·15	0·18§	–0·7, 0·6	2·15**||	0·9, 3·1	1·60||	0·7, 2·7
Tomatoes (150 kJ)	0·41	0·30	0·48	0·53	0·08	–0·2, 0·3	0·41	0·32	0·33	0·35	–0·08	–0·3, 0·2	0·16	–0·2, 0·5	0·16	–0·1, 0·5
Other red and orange vegetables‡ (150 kJ)	0·44	0·67	1·92	2·74	1·93**§	1·0, 3·3	0·53	1·09	0·55	1·06	0·24§	–0·6, 0·7	1·90**||	0·6, 3·2	1·31**||	0·6, 2·7
Starchy vegetables (250 kJ)	0·96	0·93	0·19	0·40	–0·77**	–1·3, –0·3	1·43	1·86	0·79	1·16	–0·64*	–1·2, –0·1	–0·12	–0·8, 0·6	–0·59*	–1·2, 0·0
Potatoes (250 kJ)	0·73	0·80	0·18	0·39	–0·55*	–0·1, –0·1	1·26	1·89	0·72	1·15	–0·54	–1·1, 0·0	–0·01	–0·7, 0·7	–0·54*	–1·07, –0·01
Other starchy vegetables (250 kJ)‡	0·18	0·34	0·00	0·06	–0·17**§	–0·2, –0·1	0·00	0·18	0·01	0·10	0·0§	–0·2, 0·0	–0·04||	–0·2, 0·0	0·00||	–0·03, 0·00
Starchy vegetable percentage	27·86	26·02	4·29	9·82	–23·58**	–36·6, –10·6	29·90	26·65	18·70	25·70	–11·21	–22·7, 0·3	–12·37	–29·8, 5·0	–14·41**	–28·1, –0·7
Legumes (350 kJ)‡	0·00	0·15	0·00	0·03	–0·06*§	–0·1, 0·0	0·00	0·10	0·004	0·91	0·01§	–0·1, 0·5	–0·05||	–0·3, 0·0	–0·04*||	–0·4, 0·0
Other vegetables (100 kJ)‡	0·96	0·72	1·64	1·49	0·68*§	0·1, 1·3	1·27	0·67	1·27	1·51	0·21§	–0·3, 0·5	0·55||	–0·3, 1·5	0·43||	–0·2, 1·1
Total protein foods‡	2·59	1·15	3·91	1·58	1·49**§	0·9, 2·1	2·61	1·71	2·56	1·73	–0·22§	–0·90, 0·6	1·72**||	0·8, 2·7	1·42**||	0·6, 2·3
Red meat (20 g Ptn)‡	0·50	0·74	1·36	1·36	0·65**§	0·2, 1·1	0·32	1·22	0·00	0·59	–0·32§	–0·9, 0·0	1·02**||	0·3, 1·7	0·92**||	0·6, 1·5
Poultry (23 g Ptn)‡	0·36	0·77	0·50	0·96	0·14§	–0·1, 0·5	0·69	0·98	0·52	0·82	0·07§	–0·4, 0·7	0·13||	–0·4, 0·7	–0·11||	–0·4, 0·3
Eggs (13 g Ptn)‡	0·36	0·77	0·52	0·52	0·22*§	0·1, 0·4	0·05	0·37	0·28	0·44	0·14§	0·0, 0·3	0·07||	–0·2, 0·3	0·26*||	0·04, 0·5
Processed meat (20 g Ptn)‡	0·08	0·18	0·00	0·28	–0·01§	–0·1, 0·0	0·00	0·24	0·00	0·10	–0·03§	–0·1, 0·0	0·03||	0·0, 0·1	0·00||	0·00, 0·1
Organ meat (20 g Ptn)‡	0·00	0·00	0·0	0·0	0·0§	0·0, 0·0	0·00	0·00	0·0	0·00	0·0§	0·0, 0·0	0·00||	0·0, 0·0	0·00||	0·00, 0·00
Seafood high *n*-3 (23 g Ptn)‡	0·00	0·31	0·13	0·44	0·01§	0·0, 0·2	0·00	0·09	0·02	0·30	0·05§	0·0, 0·2	0·00||	–0·1, 0·1	0·00||	–0·2, 0·1
Seafood low *n*-3 (23 g Ptn)‡	0·00	0·25	0·05	0·49	0·0§	–0·1, 0·2	0·09	0·32	0·00	0·42	0·02§	–0·1, 0·2	0·00||	–0·2, 0·2	0·00||	–0·1, 0·2
Nuts and seeds (30 g Ptn)‡	0·31	0·71	0·83	1·16	0·22§	–0·1, 0·6	0·54	0·47	0·45	0·69	–0·25§	–0·5, 0·1	0·45*||	0·0, 0·9	0·07||	–0·3, 0·4
Legumes protein (9 g Ptn)‡	0·00	0·08	0·00	0·03	–0·03*§	0·1, 0·0	0·00	0·08	0·003	0·51	0·01§	0·0, 0·3	–0·02*||	–0·2, 0·0	–0·00*||	–0·2, 0·0
Soya products (9 g Ptn)‡	0·00	0·00	0·00	0·00	0·0§	0·0, 0·0	0·0	0·00	0·00	0·00	0·0§	0·0, 0·0	0·0||	0·0, 0·0	0·00||	0·0, 0·0
Total dairy products	1·57	0·71	0·09	0·19	–1·48**	–1·8, –1·2	1·93	0·97	1·54	0·59	–0·39	–0·9, 0·1	–1·09**	–1·6, –0·5	–1·45**	–1·8, –1·1
Milk (300 mg Ca)	0·84	0·48	0·08	0·17	–0·76**	–1·0, –0·6	1·14	0·65	0·84	0·51	–0·30*	–0·5, –0·1	–0·46**	–0·8, –0·2	–0·76**	–1·0, –0·5
Cheese (300 mg Ca)‡	0·49	0·51	0·00	0·00	–0·47**§	–0·7, –0·3	0·41	0·62	0·30	0·45	–0·08§	–0·3, 0·2	–0·40**||	–0·7, –0·2	–0·30**||	–0·4, –0·2
Yogurt (300 mg Ca)‡	0·07	0·40	0·0	0·0	–0·19**§	–0·3, –0·03	0·19	0·39	0·18	0·40	0·01§	–0·1, 0·1	–0·16||	–0·3, –0·1	–0·18**||	–0·3, 0·0
Milk alternatives (300 mg Ca)‡	0·00	0·00	0·0	0·0	0·0§	0·0, 0·0	0·00	0·00	0·00	0·00	0·0§	0·0, 0·0	–0·00||	0·0, 0·0	0·00||	0·0, 0·0
Oil equivalents (4·6 g, 1 tsp)‡	6·81	3·04	6·19	6·53	0·68§	–1·5, 2·8	7·89	5·23	7·10	4·03	–1·48§	–3·9, 1·3	1·93||	–1·1, 5·3	–0·44||	–2·5, 2·6
Solid fat equivalents (4·6 g, 1 tsp)‡	7·50	4·39	5·23	3·42	–2·00**§	–3·7, –0·7	8·82	4·11	5·38	2·85	–3·03*§	–4·6, –0·7	1·09||	–1·8, 3·1	–0·44||	–2·0, 1·0
Added sugars (4·2 g, 1 tsp)	8·61	7·32	2·97	3·71	–5·64**	–8·4, –2·9	6·34	4·52	3·83	2·38	–2·50*	–4·4, –0·6	–3·13	–6·4, 0·1	–0·86	–3·0, 1·2
Added sugars (kJ)	578·39	492·18	199·64	248·98	–378·8**	–561·7, –195·8	425·78	303·85	257·53	160·19	–168·25*	–267·7, –38·8	–210·50	–427·9, 6·9	–57·89	–198·7, 82·9
Added sugars (% of energy)	6·55	4·45	3·43	4·01	–3·12**	–5·0, –1·2	4·93	2·96	4·00	2·48	–0·93	–2·4, 0·5	–2·19	–4·5, 0·1	–0·57	–2·8, 1·7

AGHE, Australian Guide to Healthy Eating; Ptn, protein; tsp, teaspoon.*P<0·05, **P<0·01.

† Food group serve sizes as used by FoodWorks Professional^(^ ^26^ ^)^. Normally distributed data were assessed using paired *t* tests and independent *t* tests.

‡ Non-normally distributed data. Results are shown as medians and IQR.

§ Non-normally distributed data were assessed using the Wilcoxon signed rank matched pair test; results are shown as median differences. The corresponding 95 % CI were calculated using the Hodges–Lehmann estimator in SPSS.

|| Non-normally distributed data were assessed using the Mann–Whitney *U* test. Note that the 95 % CI corresponding to the Mann–Whitney *U* test are given for the median differences and were determined using the Hodges–Lehmann estimator in SPSS.

### Fibre

There were no significant differences between groups in total dietary fibre, soluble or insoluble fibre intake (Table 2). Post-intervention, the Paleolithic compared with AGHE group had a significantly lower intake of total starch (–61·4 g/d, *P* <0·01) and RS, at both minimum (–1·18 g/d, *P* <0·01) and maximum (–3·54 g/d, *P* <0·05) estimated intakes.

**Table 2:** Total dietary fibre, insoluble and soluble fibre and resistant starch intake pre- and post-intervention and differences between groups(Mean values and standard deviations; medians and interquartile ranges (IQR); medians and 95 % confidence intervals)

Dietary variable	Paleolithic (*n* 22)						AGHE (*n* 17)									
	Pre		Post		Change within group		Pre		Post		Change within group		Difference between groups (change)		Difference between groups (post-intervention)	
	Mean	sd	Mean	sd	Median	95 % CI	Mean	sd	Mean	sd	Median	95 % CI	Median	95 % CI	Median	95 % CI
Total dietary fibre (g)	22·77	6·50	22·93	7·19	0·15	–3·62, 3·93	23·84	6·45	23·63	10·15	–0·21	–5·25, 4·84	0·36	5·60, 6·32	–0·70	–6··32, 4·92
Soluble dietary fibre (g)	8·29	2·37	8·15	2·70	–0·13	–1·47, 1·21	9·88	3·53	8·18	3·34	–1·70	–3·69, 0·29	1·57	–0·66, 3·80	–0·02	–1·98, 1·93
Insoluble dietary fibre (g)	12·81	4·37	12·23	4·44	–0·58	–2·93, 1·78	12·99	4·16	13·92	6·43	0·93	–2·47, 4·33	–1·51	–5·38, 2·36	–1·69	–5·22, 1·84
Total starch intake (g)	95·63	32·12	23·63	17·84	–72·00**	–87·19, –56·81	94·85	49·02	85·06	33·14	–9·79	–31·83, 12·26	–62·21**	–87·22, –37·20	–61·43**	–79·82, –43·04
Resistant starch (min) (g)†‡	2·56	1·22	1·39	0·95	–1·27**§	–1·83, –0·86	2·45	1·58	2·46	2·26	0·24§	–0·53, 1·05	–1·63**||	–2·52, –0·62	–1·18**||	–2·03, –0·45
Resistant starch (max) (g)†‡	7·60	3·86	6·52	4·59	–0·89§	–2·60, 0·80	8·84	6·87	9·91	9·06	1·16§	–3·24, 6·69	–2·42||	–6·69, 2·57	–3·54*||	–7·28, –0·33

AGHE, Australian Guide to Healthy Eating.

*P<0·05, **P<0·01.

† Minimum and maximum values were determined using 3-d weighed food records with the addition of resistant starch obtained from data published by Roberts *et al.* ^(^ ^13^ ^)^, Food Standards Australia New Zealand^(^ ^32^ ^)^ and Landon *et al.* ^(^ ^49^ ^)^. Normally distributed data were assessed using paired *t* tests and independent *t* tests.

‡ Non-normally distributed data. Results are shown as medians and IQR.

§ Wilcoxon signed rank matched pair test; results are shown as median differences. The corresponding 95 % CI were calculated using the Hodges–Lehmann estimator in SPSS.

|| Mann–Whitney *U* test. The corresponding 95 % CI were determined using the Hodges–Lehmann estimator in SPSS.

### Trimethylamine-*N*-oxide

There were no significant differences between groups post-intervention (Paleolithic median 6·79 (sd 12·92) μm *v*. AGHE 6·85 (sd 10·89) μm, *P* = 0·532) or change in serum TMAO concentration (Paleolithic median change 3·39 μm *v*. AGHE 1·19 μm, *P* = 0·654, effect size = 0·10). The inclusion of protein serves, solid fat, red meat, egg and grain serves in addition to estimates of RS as covariates in the ANCOVA model did not alter this finding.

## Discussion

This dietary intervention in a group of thirty-nine healthy women examined the differences in intake of core food groups and types of fibre between the Paleolithic and AGHE diets. We have reported total dietary fibre intake consistent with other studies of the Paleolithic diet^(^ ^4^ ^–^ ^6^ ^,^ ^8^ ^,^ ^10^ ^)^, however, the changes in RS intake reported here represent new findings and may have long-term health implications. Over the short-term intervention, our results indicate that fasting serum concentrations of TMAO were not influenced by the relative increase in serves of eggs and red meat consumed on a Paleolithic diet or the decrease in RS intake.

With the exclusion of grains, cereals, dairy products and legumes on the Paleolithic diet, it was anticipated that differences would occur in the intake of the protein food group, which included meats, poultry, and alternatives such as seafood, nuts and seeds and legumes. The increase we found in the protein food group was attributable to greater red meat and egg consumption, precursor foods for the formation of TMAO^(^ ^16^ ^)^. Despite the increased serves of fruits and vegetables consumed in the Paleolithic group and the maintenance of total dietary fibre intake, RS was negatively impacted by the elimination of whole grains, cereals and legumes. In the AGHE dietary intervention group, we also note no increase in consumption of legumes, which provide a good source of RS^(^ ^13^ ^)^, and may provide an area where these data can guide future interventions using the AGHE. While RS intake was low on the Paleolithic diet, RS can be obtained on a Paleolithic diet from root vegetables and bananas^(^ ^13^ ^)^.

SCFA (primarily acetate, propionate and butyrate) are products of bacterial fermentation of CHO in the colon^(^ ^34^ ^)^. Butyrate, in particular, contributes to the maintenance of colonic epithelia^(^ ^35^ ^)^. Previous research has shown that low-CHO diets decrease total faecal SCFA concentrations and total daily faecal SCFA excretion when compared with energy-restricted high-CHO diets^(^ ^36^ ^)^. Consumption of RS is known to increase the faecal output of SCFA, and epidemiological evidence suggests it is protective against colorectal cancer^(^ ^35^ ^)^. As evidence emerges on the link between diet and bacterial populations inhabiting the bowel^(^ ^36^ ^–^ ^38^ ^)^, the reduction in RS intake we have reported may also reduce colonic SCFA production, which may, in turn, increase the risk of developing non-infectious digestive disorders^(^ ^36^ ^,^ ^39^ ^)^. Bergeron *et al.* ^(^ ^25^ ^)^ recently reported that plasma TMAO concentrations were increased, in a 2-week crossover study using high CHO, with high or low RS (51 % of energy CHO (27 g RS) and 53 % CHO (<2 g RS)), compared with low CHO, with high or low RS (40 % CHO (19 g RS) and 39 % CHO (<1 g RS)). The authors reported the Hi-Maize 260 supplement used for the study comprised 41·5 % RS^(^ ^25^ ^)^. TMAO concentrations were significantly higher on the high *v*. low RS intake, but the effect was also dependent on dietary CHO intake^(^ ^25^ ^)^. The authors concluded that both dietary CHO and RS intake influence the production of TMA by colonic microbiota. However, the low-CHO diets were also much higher in fat (40 and 41 % of energy, respectively). Previous research in rats indicated that the benefits of RS supplementation were attenuated when the animals were fed a high-fat diet (40–41 % of energy)^(^ ^40^ ^)^. Therefore, a reduction in beneficial bacteria mediated by the high-fat diet may have led to the increase in blood TMAO concentrations. Without faecal biochemistry measurements, such as SCFA concentrations, it is not possible to conclude that the increase in TMAO was due to the RS supplementation alone. Diets low in CHO and whole grains also have lower content of other fermentable fibres that could influence the gut microbiota, including oligosaccharides, *β*-glucans and arabinoxylans^(^ ^41^ ^)^. A full understanding of dietary modification of TMAO concentrations can be gained with future measurement of these variables in conjunction with RS. In our short-term intervention, the reduction in RS intake and the relative increases in meat and egg consumption did not impact serum concentrations of TMAO, although we note the small effect size of the TMAO result, and our small sample size may have limited our ability to detect the differences in this area. Furthermore, participants in both dietary intervention groups decreased energy intake significantly^(^ ^3^ ^)^, which is known to impact the microbiota^(^ ^42^ ^–^ ^44^ ^)^, and may have influenced the ability of the microbiota to produce TMA. However, the link between diet, the microbiota and TMAO is not yet well understood, and larger, long-term research in this area is required. Recent epidemiological and experimental evidence has strengthened the link between red meat intake and colorectal cancer^(^ ^45^ ^–^ ^47^ ^)^. Further research examining the long-term health impacts of the Paleolithic diet is now warranted, considering that RS intake is low on the dietary pattern. Examination of TMAO concentrations with longer-term adherence to a Paleolithic diet, which includes an examination of microbiota composition is recommended, aiding further understanding of the role of dietary patterns, RS intake and the microbiota in modulating TMAO concentrations and CVD risk.

Our examination of fibre intake has highlighted limitations in the current Australian fibre data. Older methods of measuring total dietary fibre (AOAC 985.29 and 991.43) fail to capture/quantify all CHO components that constitute dietary fibre, as currently defined^(^ ^48^ ^)^. For instance, they underestimate most types of RS^(^ ^48^ ^)^. Drawing conclusions linking dietary fibre fraction intake with health outcomes is limited without having a complete understanding of dietary fibre fraction composition; thus, more research is now required to develop updated databases in this area.

Despite the increased serves of fruits and vegetables consumed in the Paleolithic group and the maintenance of total dietary fibre intake, RS was negatively impacted by the elimination of whole grains and cereals. Further research is required to determine the effect of this reduction on the gut microbiota and subsequent health outcomes; in particular, the association with serum TMAO concentrations and CVD. Furthermore, with increased understanding of the physiological responses to fibre fractions, it is critical that the Australian and New Zealand fibre databases are updated to include RS components to enable population health assessment and establish epidemiological links with chronic disease.
